# Subungual Hematoma: Insights From a Clinical Case Study

**DOI:** 10.7759/cureus.84493

**Published:** 2025-05-20

**Authors:** Ricardo Cid Puente, Ana I Diaz de León Guzmán, Laura S De León Puga, Paola V Rosales Verduzco, Amairani Tovar Garcia

**Affiliations:** 1 Department of Immunology, Biological Sciences School, Universidad Autónoma de Zacatecas, Zacatecas, MEX; 2 Department of Dermatology, Hospital Civil de Culiacán, Secretaría de Salud, Culiacán, MEX; 3 Department of Internal Medicine, Hospital Regional Pemex, Ciudad Madero, Ciudad Madero, MEX; 4 Department of Internal Medicine, Hospital Regional "Dr. Valentín Gómez Farías" Instituto de Seguridad y Servicios Sociales de los Trabajadores del Estado (ISSSTE), Zapopan, MEX; 5 Department of Internal Medicine, Hospital General de Xoco, Secretaría de Salud, Mexico City, MEX

**Keywords:** nail pigmentation, nail trauma, subungual hematoma, subungual hemorrhages, subungual lesion

## Abstract

Subungual hematoma (SH) refers to the accumulation of blood under the nail due to traumatic injury. In this article, we report the case of a 61-year-old male with a history of diabetic neuropathy (DN) who presented to our clinic with an SH on the first toe of the left foot, secondary to direct trauma while working. Due to DN, the patient did not experience pain and did not seek medical attention until he observed generalized black pigmentation on the toenail. After the initial evaluation, the hematoma was drained at the primary care clinic by trephination with an 18-gauge needle. Following the procedure, the patient showed complete remission of the pigmentation with no complications. In this article, we highlight the importance of SH, its clinical presentation, diagnostic suspicion, and appropriate treatment to avoid complications.

## Introduction

Subungual hematoma (SH) is a phenomenon characterized by the accumulation of blood beneath the nails of the hands or feet [1]. Its etiology is traumatic in most cases; however, certain predisposing factors exist, such as coagulopathies and anticoagulant therapy, which increase the risk of bleeding with minor trauma, and diabetic neuropathy (DN), a peripheral symmetric polyneuropathy that causes abnormal sensory function, resulting in loss of sensitivity in the foot and thereby making injuries more frequent [2-4]. It is a relatively common pathology in the emergency room, and its main characteristic is dark pigmentation of the nail plate and sudden, intense pain due to increased pressure between the nail bed and the nail plate [1]. There is no specific test, and its diagnosis is clinical; a good physical examination and thorough history-taking make the diagnosis in most cases. Nevertheless, a differential diagnosis with subungual melanoma (SM) should always be considered [4-7]. The treatment of SH is usually simple and mainly focuses on draining the hematoma by performing a trephination on the nail plate, which can be done at the primary care level by general practitioners or nursing staff. Surgical removal of the nail is increasingly disused, and its indications are few [8-10]. The main purpose of this article is to inform the primary care physician about a common but often underrecognized pathology, which can be treated effectively on an outpatient basis through a quick procedure such as trephination.

## Case presentation

**Figure 1:**
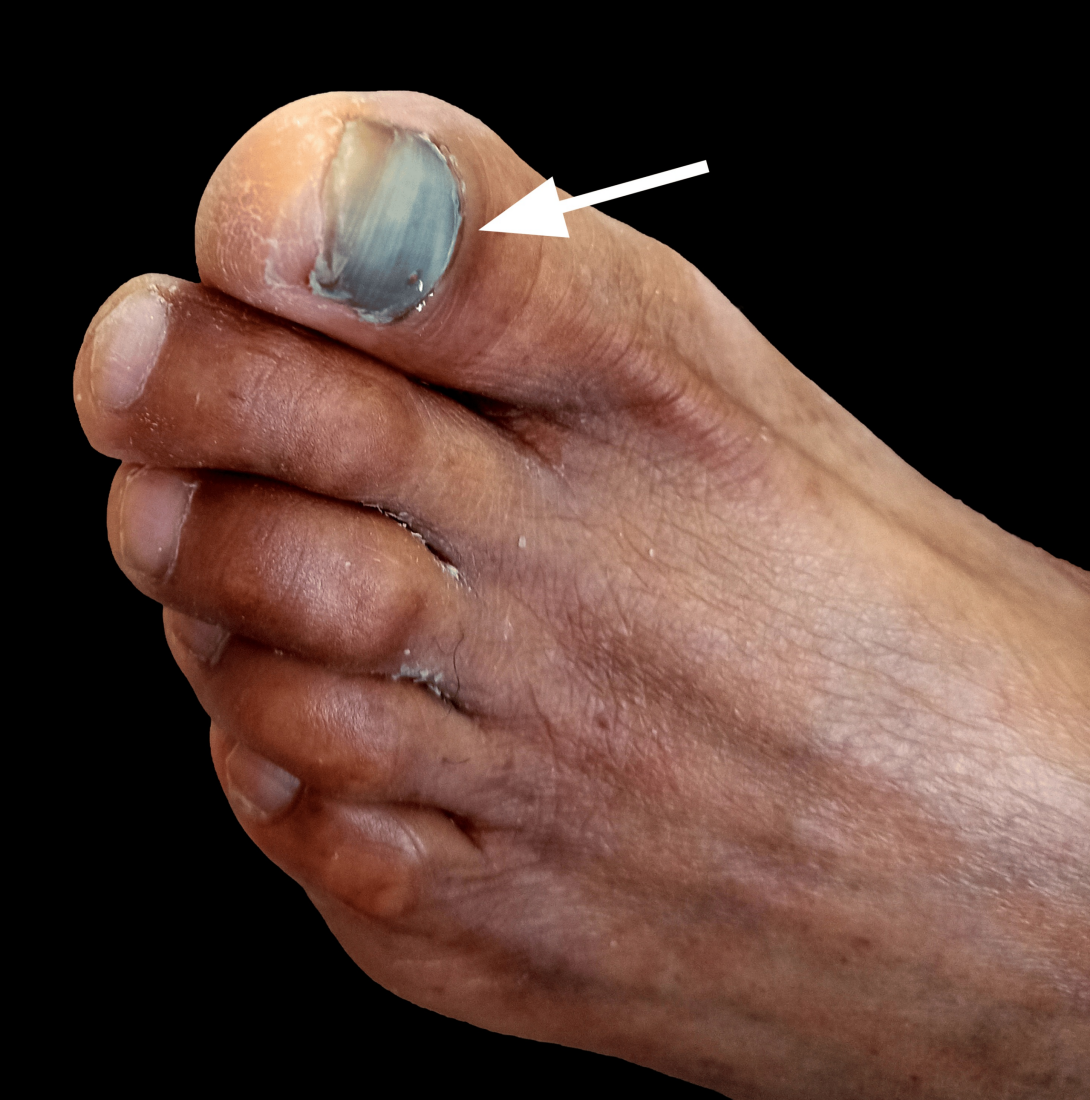
Subungual hematoma prior to drainage. The white arrow indicates dark pigmentation of the nail due to blood accumulation beneath the nail plate.

We present the case of a 61-year-old male construction worker from Zacatecas, Mexico, with no notable family history. His past medical history was significant for systemic arterial hypertension, type 2 diabetes, and long-standing DN. He was treated with dapagliflozin 10 mg daily, metformin 850 mg twice a day, telmisartan 40 mg, nifedipine 30 mg daily, and pregabalin 75 mg twice a day. The patient reported good adherence to the treatment. He attended our primary care clinic for his scheduled chronic disease check-up appointment, during which he mentioned having noticed dark pigmentation on the entire nail of the first toe of his left foot for the past 14 days. He associated this with a probable fungal infection, for which he had self-medicated with itraconazole cream, without improvement. Upon physical examination, dermatosis was identified on the first nail plate of the left foot, presenting with pachyonychia, distal xanthonychia, and diffuse black pigmentation of the toenail with irregular borders that did not blanch under pressure and was not associated with pain. No alterations were noted in the remaining toes (Figure 1). A direct interrogation was conducted, during which the patient reported having hit his left foot with a brick while at work approximately two weeks prior.

**Figure 2:**
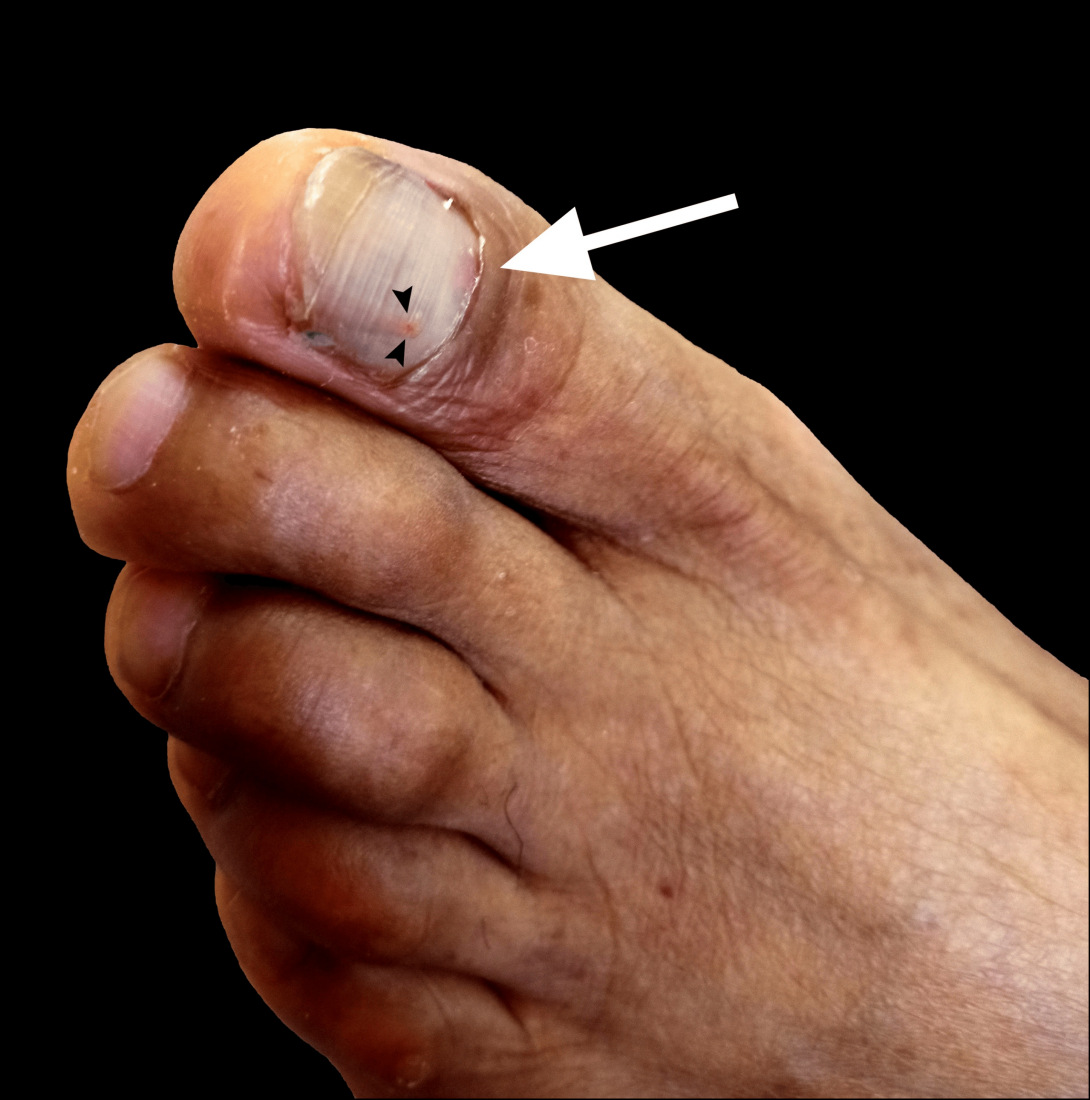
Subungual hematoma post-drainage. The white arrow shows clearing of the nail after the hematoma was drained. The black arrowheads indicate the site where trephination was performed.

Based on clinical characteristics and the patient’s history of trauma, an SH was suspected. The hematoma was confirmed upon drainage via trephination of the toenail using an 18-gauge needle (Video 1). Post-procedure, there was noticeable clearing of the nail with no complications (Figure 2).

**Video 1:** Subungual hematoma drainage. This video presents the drainage of a subungual hematoma following nail trephination using an 18-gauge needle.

## Discussion

SH is the accumulation of blood beneath the nail, either on the feet or hands. This phenomenon predominantly occurs due to direct trauma to the nail bed. Clinically, a change in the color of the nail bed to brown or black is observed. Typically, the trauma is both painful and evident, with SH potentially appearing immediately or several hours post-injury. If left untreated or inadequately managed, it may lead to chronic deformities of the nail bed, nail plate, and fingertip, possibly resulting in long-term functional deficits [1,2]. However, in instances where nail pigmentation changes cannot be attributed to clear physical trauma, it is advisable to conduct a thorough examination of the lesion. An alternative explanation for cases where the clinical presentation strongly indicates SH but the patient denies any history of trauma is Munchausen syndrome. This condition involves patients inflicting injuries on themselves to obtain medical attention. Typically, these patients have a history of numerous hospitalizations, and their initial accounts are often exaggerated and lack credibility [11]. Additionally, it is important to inquire about potential predisposing factors for SH, such as coagulation disorders, anticoagulant therapy, peripheral vascular disease, and DN [2,4]. The latter is of vital importance because, in these patients, the loss of sensitivity in the feet masks the clinical picture and makes diagnosis difficult, as they do not present the intense pain characteristic of SH and usually do not seek medical attention until they notice the change in nail color, as in the case of our patient.

Despite being a common lesion, its incidence and prevalence are difficult to establish because it is rarely reported. Nonetheless, its presentation is very common in emergency rooms and primary care clinics. It can be observed in all age groups without gender distinction. Its incidence is higher in warm climates due to the frequent use of sandals and in adults, directly related to occupational and recreational activities such as construction work and contact sports [2].

Throbbing pain is the first clinical sign identified by the patient and is due to the accumulation of blood between the nail plate and the nail bed, which increases the pressure between them and results in intense pain. In severe cases, the increased pressure can cause the nail plate to separate from the nail bed [1]. Clinically, it manifests as a reddish to black pigmentation of the nail, which may be partial or generalized and does not blanch with pressure. Typically, physical examination, onychoscopy, and clinical history are sufficient to diagnose SH [4]. On onychoscopy, one can observe irregular dark pigmentation that progresses distally with nail growth and should be monitored until complete resolution. Additionally, this condition may be associated with nail deformity or leukonychia [5].

The differential diagnosis of pigmented nail lesions is extensive and can be of melanocytic or non-melanocytic origin. Generally, three main causes can be identified: increased melanin production (onychomycosis, Addison's disease, drugs, chronic trauma), melanocyte hyperplasia (SM, lentigo, nevus), and non-melanocytic pigments (SH, cosmetics, nicotine, hydroxyurea, Pseudomonas infection) [5,6]. It is imperative to consider a differential diagnosis with SM in all cases of pigmented nail lesions due to its aggressive and potentially fatal progression. Key considerations include the elevated frequency among Asian and African American populations, its prevalent onset during the fifth to seventh decades of life, its higher incidence in males, and the absence of correlation with sun exposure. Clinically, SM typically affects a single digit, most commonly the first toe, and is characterized by rapidly expanding heterogeneous black pigmentation with irregular borders that may extend to the periungual skin, known as Hutchinson's sign. Pain, recurrent bleeding, and ulcerations are indicative of advanced disease stages. Early onset of the lesion, involvement of multiple digits, and slow progression are typically associated with benign pathologies [5,7]. A practical way to suspect SM is through the “ABCDEF” criteria described by Levit EK et al. (Table 1) [12].

**Table 1:** “ABCDEF” criteria for the clinical detection of subungual melanoma.

Criteria	Definition
A	Age (40-69 years); African-American, Asian, or Native American ethnicity
B	Brown-Black pigment; breadth >3 mm; band (longitudinal melanonychia); irregular borders
C	Change in the nail band (e.g., rapid growth)
D	Digit involved (most commonly thumb, hallux, or index finger)
E	Extension (Hutchinson’s sign)
F	Family or personal history of previous melanoma

This patient's case exemplifies a challenging diagnosis due to numerous similarities with SM. However, the critical factors supporting the diagnosis of SH included the history of trauma, homogenous pigmentation of the nail, and the absence of Hutchinson's sign.

Conventionally, treatment was based on the size of the hematoma: if it covered more than 50%, or more than 25% in the presence of a distal phalanx fracture, the nail plate was surgically removed. Conversely, more recent studies have shown that draining the hematoma through trephination of the nail provides similar or better outcomes compared to nail removal. Expectant treatment should only be considered in cases where there is no fracture and the SH does not cause any pain [8,9].

Trephination is conducted at the base of the nail using a sterile 18-gauge needle, which is maneuvered in a circular motion while applying consistent pressure to the nail. Alternative techniques include using a heated paperclip tip or an electrocautery device [9,10].

In this case, the patient presented with an SH involving more than 50% of the toenail, which would typically require surgical management with nail removal. However, adequate drainage and resolution were achieved without complications using the nail trephination technique.

## Conclusions

SH is a common nail condition that should be primarily suspected in patients with a history of trauma. It is characterized by reddish to black nail pigmentation and intense throbbing pain. The diagnosis is essentially clinical, as there is no specific test for its detection. Careful consideration should be given to patients with DN, as their clinical presentation may be less apparent due to decreased sensitivity. It is imperative for primary healthcare professionals to rapidly identify SH and educate patients on early recognition to prevent long-term complications, especially in those with DN. However, if the clinical picture is unclear, a differential diagnosis with SM should be considered. In most cases, treatment involves trephination to drain the hematoma and alleviate symptoms by decompressing the area between the nail plate and the nail bed. This procedure is rapid, straightforward, and has largely replaced surgical intervention. Therefore, it is recommended to perform trephination promptly when there is a high suspicion of SH.
